# Aqua­bis­(5-butyl­pyridine-2-carboxyl­ato)zinc monohydrate

**DOI:** 10.1107/S1600536811021623

**Published:** 2011-06-11

**Authors:** Yi-Wen Tao, Yun Wang

**Affiliations:** a School of Basic Science, Guangzhou Medical College, Guangzhou 510182, People’s Republic of China; b Guangdong Institute for Drug Control, Guangzhou 510180, People’s Republic of China

## Abstract

In the title complex, [Zn(C_10_H_12_NO_2_)_2_(H_2_O)]·H_2_O, the Zn^II^ ion is coordinated by two N and two O atoms from two 5-*n*-butyl­pyridine-2-carboxyl­ato ligands and one O atom from a water mol­ecule in a distorted square-pyramidal geometry. In the crystal, inter­molecular O—H⋯O hydrogen bonds link mol­ecules into a two-dimensional supramolecular structure parallel to (001).

## Related literature

For related structures, see: Pons *et al.* (2004 ▶); Yoshikawa *et al.* (2002 ▶); Qin *et al.* (2007 ▶); He *et al.* (2007 ▶).
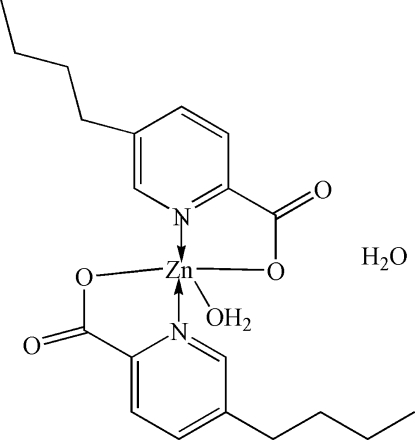


## Experimental

### 

#### Crystal data

[Zn(C_20_H_12_NO_2_)_2_(H_2_O)]·H_2_O
*M* *_r_* = 457.81
Triclinic, 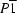

*a* = 9.6949 (13) Å
*b* = 10.0054 (14) Å
*c* = 11.9249 (17) Å
α = 97.799 (2)°
β = 91.533 (2)°
γ = 113.464 (2)°
*V* = 1047.1 (3) Å^3^
*Z* = 2
Mo *K*α radiation
μ = 1.21 mm^−1^
*T* = 293 K
0.48 × 0.42 × 0.15 mm

#### Data collection

Bruker APEXII CCD area-detector diffractometer
Absorption correction: multi-scan (*SADABS*; Sheldrick, 2008*a* ▶) *T*_min_ = 0.594, *T*_max_ = 0.839
7770 measured reflections
3847 independent reflections
3410 reflections with *I* > 2σ(*I*)
*R*_int_ = 0.017

#### Refinement

*R*[*F* ^2^ > 2σ(*F* ^2^)] = 0.027
*wR*(*F* ^2^) = 0.075
*S* = 1.06
3847 reflections
264 parameters
H-atom parameters constrained
Δρ_max_ = 0.47 e Å^−3^
Δρ_min_ = −0.29 e Å^−3^

### 

Data collection: *APEX2* (Bruker, 2004 ▶); cell refinement: *SAINT-Plus* (Bruker, 2001 ▶); data reduction: *SAINT-Plus*; program(s) used to solve structure: *SHELXS97* (Sheldrick, 2008*b* ▶); program(s) used to refine structure: *SHELXL97* (Sheldrick, 2008*b* ▶); molecular graphics: *SHELXTL* (Sheldrick, 2008*b* ▶); software used to prepare material for publication: *SHELXTL*.

## Supplementary Material

Crystal structure: contains datablock(s) I, global. DOI: 10.1107/S1600536811021623/hg5046sup1.cif

Structure factors: contains datablock(s) I. DOI: 10.1107/S1600536811021623/hg5046Isup2.hkl

Additional supplementary materials:  crystallographic information; 3D view; checkCIF report

## Figures and Tables

**Table 1:** Hydrogen-bond geometry (Å, °)

*D*—H⋯*A*	*D*—H	H⋯*A*	*D*⋯*A*	*D*—H⋯*A*
O2*W*—H2*B*⋯O4	0.85	1.94	2.780 (2)	172
O2*W*—H2*A*⋯O4^i^	0.85	2.02	2.849 (2)	165
O1*W*—H1*B*⋯O2*W*^ii^	0.84	1.80	2.638 (2)	175
O1*W*—H1*A*⋯O2^iii^	0.85	1.79	2.636 (2)	174

Symmetry codes: (i) 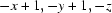; (ii) 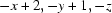; (iii) 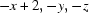.

**Fig. 1.**
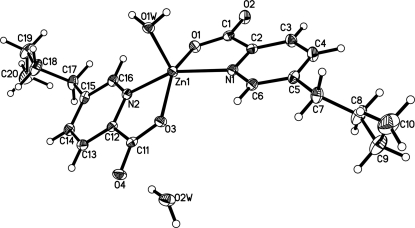
The molecular structure of the title complex with the atom-labelling scheme. Displacement ellipsoids are drawn at the 30% probability level.

## supplementary crystallographic information

### Comment

As known for a long time, pyridine-2-carboxylato ion and its derivatives are good chelating ligands for many transition metals(Pons, *et al.*, 2004; Yoshikawa, *et al.*, 2002; Qin *et al.*, 2007; He *et al.*, 2007). Here, we report a new zinc complex based on 5-n-butylpyridine-2-carboxylate.

The crystal structure of the title compound,(I), is shown in Fig. 1. The coordination sphere of the Zn^II^ ion should be described as a distorted square-pyramidal, in which the four basal positions are occupied by two N atoms and two O atoms from two 5-n-butylpyridine-2-carboxylato ligands, and the fifth apical site is occupied by the O atom of the coordinated solvent water molecule. The Zn(II) ion is out of the plane formed by N_2_O_2_ unit 0.504 (8)Å towards the Zn—O_water_ bond. The average Zn—N, Zn—O_ligand_, and the Zn—O_water_ are 2.094, 2.043, and 2.044Å, respectively, in agreement with the corresponding distances found in other Zn complexes containing similar ligands (Qin *et al.*, 2007; He *et al.*, 2007). It was noting that the title complex was linked into a two-dimensional supermolecular structure under the help of the intermolecular O—H···O hydrogen bonds interactions.

### Experimental

The title complex was prepared by the addition of a stiochiometric amount to zinc acetate dehydrate (0.054 mmol) to a hot aqueous solution (5 ml) of fusaric acid (30 mmol) which was extracted from the endophytic fungus phomopsis *sp*. from the mangrove tree on the South China Sea coast. The resulting solution was filtered, and colorless crystals were obtained by slow evaporation of a methanol-water solution (90:10 *v*/*v*) at room temperature over several days.

### Refinement

All the H atoms bonded to the C atoms were placed using the HFIX commands in *SHELXL-97*, with C—H distances of 0.95, 0.98 and 0.99 Å, and were allowed for as riding atoms with *U*_iso_(H) = 1.2 or 1.5*U*_eq_(C). For the H atom of the water molecule, they were found from difference Fourier maps with the O—H bond length restrained to 0.85 Å.

### Crystal data

**Table:**

[Zn(C_20_H_12_NO_2_)_2_(H_2_O)]·H_2_O	*Z* = 2
*M**_r_* = 457.81	*F*(000) = 480
Triclinic, *P*1	*D*_x_ = 1.452 Mg m^−^^3^
Hall symbol: -P 1	Mo *K*α radiation, λ = 0.71073 Å
*a* = 9.6949 (13) Å	Cell parameters from 5809 reflections
*b* = 10.0054 (14) Å	θ = 2.3–27.0°
*c* = 11.9249 (17) Å	µ = 1.21 mm^−^^1^
α = 97.799 (2)°	*T* = 293 K
β = 91.533 (2)°	Prism, colorless
γ = 113.464 (2)°	0.48 × 0.42 × 0.15 mm
*V* = 1047.1 (3) Å^3^	

### Data collection

**Table:**

Bruker APEXII CCD area-detector diffractometer	3847 independent reflections
Radiation source: fine-focus sealed tube	3410 reflections with *I* > 2σ(*I*)
graphite	*R*_int_ = 0.017
phi and ω scans	θ_max_ = 25.5°, θ_min_ = 1.7°
Absorption correction: multi-scan (*SADABS*; Sheldrick, 2008*a*)	*h* = −11→11
*T*_min_ = 0.594, *T*_max_ = 0.839	*k* = −12→12
7770 measured reflections	*l* = −14→14

### Refinement

**Table:**

Refinement on *F*^2^	Primary atom site location: structure-invariant direct methods
Least-squares matrix: full	Secondary atom site location: difference Fourier map
*R*[*F*^2^ > 2σ(*F*^2^)] = 0.027	Hydrogen site location: inferred from neighbouring sites
*wR*(*F*^2^) = 0.075	H-atom parameters constrained
*S* = 1.06	*w* = 1/[σ^2^(*F*_o_^2^) + (0.0384*P*)^2^ + 0.5567*P*] where *P* = (*F*_o_^2^ + 2*F*_c_^2^)/3
3847 reflections	(Δ/σ)_max_ = 0.008
264 parameters	Δρ_max_ = 0.47 e Å^−^^3^
0 restraints	Δρ_min_ = −0.29 e Å^−^^3^

### Special details

**Table:**

Geometry. All e.s.d.'s (except the e.s.d. in the dihedral angle between two l.s. planes) are estimated using the full covariance matrix. The cell e.s.d.'s are taken into account individually in the estimation of e.s.d.'s in distances, angles and torsion angles; correlations between e.s.d.'s in cell parameters are only used when they are defined by crystal symmetry. An approximate (isotropic) treatment of cell e.s.d.'s is used for estimating e.s.d.'s involving l.s. planes.
Refinement. Refinement of *F*^2^ against ALL reflections. The weighted *R*-factor *wR* and goodness of fit *S* are based on *F*^2^, conventional *R*-factors *R* are based on *F*, with *F* set to zero for negative *F*^2^. The threshold expression of *F*^2^ > σ(*F*^2^) is used only for calculating *R*-factors(gt) *etc*. and is not relevant to the choice of reflections for refinement. *R*-factors based on *F*^2^ are statistically about twice as large as those based on *F*, and *R*- factors based on ALL data will be even larger.

### Fractional atomic coordinates and isotropic or equivalent isotropic displacement parameters (Å^2^)

**Table:**

	*x*	*y*	*z*	*U*_iso_*/*U*_eq_	
C1	0.9784 (2)	0.0833 (2)	0.17910 (17)	0.0249 (4)	
C2	1.0361 (2)	0.2469 (2)	0.22551 (17)	0.0239 (4)	
C3	1.1290 (3)	0.3107 (2)	0.32451 (18)	0.0311 (5)	
H3	1.1603	0.2528	0.3674	0.037*	
C4	1.1761 (3)	0.4611 (2)	0.36059 (19)	0.0341 (5)	
H4	1.2413	0.5071	0.4282	0.041*	
C5	1.1283 (2)	0.5447 (2)	0.29835 (17)	0.0275 (4)	
C6	1.0337 (2)	0.4707 (2)	0.19940 (17)	0.0250 (4)	
H6	0.9991	0.5254	0.1555	0.030*	
C7	1.1750 (3)	0.7081 (2)	0.33348 (18)	0.0336 (5)	
H7A	1.1100	0.7396	0.2878	0.040*	
H7B	1.2801	0.7601	0.3147	0.040*	
C8	1.1666 (4)	0.7566 (3)	0.4578 (2)	0.0496 (7)	
H8A	1.2515	0.7516	0.5025	0.059*	
H8B	1.0716	0.6866	0.4823	0.059*	
C9	1.1725 (4)	0.9123 (3)	0.4849 (2)	0.0544 (7)	
H9A	1.0839	0.9153	0.4439	0.065*	
H9B	1.1639	0.9341	0.5673	0.065*	
C10	1.3102 (4)	1.0292 (3)	0.4550 (3)	0.0681 (9)	
H10A	1.3987	1.0303	0.4976	0.102*	
H10B	1.3044	1.1251	0.4743	0.102*	
H10C	1.3193	1.0100	0.3734	0.102*	
C11	0.6412 (2)	0.2796 (2)	−0.07353 (16)	0.0238 (4)	
C12	0.5820 (2)	0.1149 (2)	−0.11734 (16)	0.0225 (4)	
C13	0.4469 (2)	0.0367 (2)	−0.18239 (18)	0.0285 (5)	
H13	0.3866	0.0852	−0.2049	0.034*	
C14	0.4004 (2)	−0.1145 (2)	−0.21448 (18)	0.0303 (5)	
H14	0.3074	−0.1704	−0.2593	0.036*	
C15	0.4900 (3)	−0.1841 (2)	−0.18108 (17)	0.0281 (5)	
C16	0.6254 (2)	−0.0962 (2)	−0.11735 (17)	0.0264 (4)	
H16	0.6890	−0.1414	−0.0949	0.032*	
C17	0.4419 (3)	−0.3486 (2)	−0.21017 (18)	0.0348 (5)	
H17A	0.5116	−0.3770	−0.1667	0.042*	
H17B	0.3397	−0.3986	−0.1853	0.042*	
C18	0.4389 (4)	−0.4036 (3)	−0.3335 (2)	0.0471 (7)	
H18A	0.5387	−0.3483	−0.3602	0.057*	
H18B	0.3629	−0.3829	−0.3767	0.057*	
C19	0.4019 (3)	−0.5685 (3)	−0.3597 (2)	0.0483 (7)	
H19A	0.4110	−0.5946	−0.4414	0.058*	
H19B	0.4777	−0.5890	−0.3161	0.058*	
C20	0.2495 (4)	−0.6646 (3)	−0.3326 (3)	0.0684 (10)	
H20A	0.2403	−0.6422	−0.2512	0.103*	
H20B	0.2340	−0.7682	−0.3520	0.103*	
H20C	0.1733	−0.6469	−0.3765	0.103*	
N1	0.98977 (19)	0.32660 (18)	0.16371 (14)	0.0230 (4)	
N2	0.67075 (19)	0.04994 (18)	−0.08590 (13)	0.0228 (3)	
O1	0.88860 (16)	0.03799 (15)	0.09036 (12)	0.0277 (3)	
O2	1.02181 (19)	0.00693 (17)	0.23255 (13)	0.0351 (4)	
O3	0.76689 (16)	0.33710 (15)	−0.01438 (12)	0.0262 (3)	
O4	0.56258 (16)	0.34586 (16)	−0.09763 (13)	0.0319 (3)	
O1W	1.02425 (16)	0.24272 (15)	−0.09978 (12)	0.0253 (3)	
H1A	1.0153	0.1655	−0.1447	0.038*	
H1B	1.1118	0.2841	−0.0657	0.038*	
O2W	0.69815 (17)	0.64141 (17)	0.00070 (14)	0.0364 (4)	
H2A	0.6306	0.6516	0.0409	0.055*	
H2B	0.6653	0.5519	−0.0315	0.055*	
Zn1	0.86873 (3)	0.19830 (2)	0.010473 (19)	0.02289 (9)	

### Atomic displacement parameters (Å^2^)

**Table:**

	*U*^11^	*U*^22^	*U*^33^	*U*^12^	*U*^13^	*U*^23^
C1	0.0274 (11)	0.0232 (10)	0.0270 (10)	0.0130 (9)	0.0052 (9)	0.0040 (8)
C2	0.0226 (10)	0.0245 (10)	0.0262 (10)	0.0113 (9)	0.0042 (8)	0.0036 (8)
C3	0.0358 (12)	0.0285 (11)	0.0303 (11)	0.0154 (10)	−0.0039 (9)	0.0032 (9)
C4	0.0394 (13)	0.0308 (12)	0.0278 (11)	0.0121 (10)	−0.0068 (10)	−0.0004 (9)
C5	0.0316 (11)	0.0231 (10)	0.0253 (10)	0.0090 (9)	0.0039 (9)	0.0015 (8)
C6	0.0281 (11)	0.0225 (10)	0.0252 (10)	0.0110 (9)	0.0035 (8)	0.0035 (8)
C7	0.0424 (13)	0.0221 (11)	0.0295 (11)	0.0072 (10)	−0.0006 (10)	0.0013 (9)
C8	0.085 (2)	0.0331 (13)	0.0338 (13)	0.0273 (14)	0.0130 (13)	0.0042 (10)
C9	0.085 (2)	0.0381 (15)	0.0421 (15)	0.0281 (15)	0.0152 (14)	0.0021 (11)
C10	0.073 (2)	0.0339 (15)	0.089 (2)	0.0174 (15)	0.0115 (18)	−0.0066 (15)
C11	0.0247 (10)	0.0241 (10)	0.0238 (10)	0.0115 (9)	0.0061 (8)	0.0019 (8)
C12	0.0224 (10)	0.0227 (10)	0.0220 (10)	0.0088 (8)	0.0053 (8)	0.0025 (8)
C13	0.0252 (11)	0.0319 (12)	0.0285 (11)	0.0128 (9)	0.0025 (9)	0.0013 (9)
C14	0.0246 (11)	0.0301 (11)	0.0272 (11)	0.0037 (9)	0.0008 (9)	−0.0017 (9)
C15	0.0349 (12)	0.0232 (10)	0.0208 (10)	0.0065 (9)	0.0046 (9)	0.0014 (8)
C16	0.0320 (11)	0.0230 (10)	0.0236 (10)	0.0105 (9)	0.0039 (9)	0.0039 (8)
C17	0.0450 (14)	0.0218 (11)	0.0283 (11)	0.0055 (10)	−0.0014 (10)	0.0005 (9)
C18	0.077 (2)	0.0301 (13)	0.0324 (13)	0.0203 (13)	0.0135 (12)	0.0027 (10)
C19	0.0670 (18)	0.0298 (13)	0.0418 (14)	0.0143 (13)	0.0186 (13)	−0.0012 (10)
C20	0.068 (2)	0.0414 (16)	0.069 (2)	0.0008 (15)	0.0174 (17)	−0.0152 (14)
N1	0.0237 (9)	0.0204 (8)	0.0241 (8)	0.0085 (7)	0.0012 (7)	0.0018 (7)
N2	0.0255 (9)	0.0207 (8)	0.0210 (8)	0.0085 (7)	0.0021 (7)	0.0018 (6)
O1	0.0304 (8)	0.0211 (7)	0.0310 (8)	0.0107 (6)	−0.0030 (6)	0.0020 (6)
O2	0.0506 (10)	0.0279 (8)	0.0316 (8)	0.0223 (8)	−0.0051 (7)	0.0020 (6)
O3	0.0234 (7)	0.0208 (7)	0.0327 (8)	0.0098 (6)	−0.0020 (6)	−0.0023 (6)
O4	0.0277 (8)	0.0270 (8)	0.0431 (9)	0.0161 (7)	−0.0029 (7)	−0.0016 (7)
O1W	0.0243 (7)	0.0229 (7)	0.0296 (7)	0.0116 (6)	0.0011 (6)	0.0012 (6)
O2W	0.0281 (8)	0.0241 (8)	0.0546 (10)	0.0112 (7)	−0.0004 (7)	−0.0025 (7)
Zn1	0.02298 (14)	0.01954 (13)	0.02499 (14)	0.00872 (10)	−0.00164 (9)	0.00013 (9)

### Geometric parameters (Å, °)

**Table:**

C1—O2	1.241 (2)	C13—C14	1.389 (3)
C1—O1	1.261 (3)	C13—H13	0.9500
C1—C2	1.519 (3)	C14—C15	1.391 (3)
C2—N1	1.345 (3)	C14—H14	0.9500
C2—C3	1.376 (3)	C15—C16	1.386 (3)
C3—C4	1.388 (3)	C15—C17	1.508 (3)
C3—H3	0.9500	C16—N2	1.342 (3)
C4—C5	1.387 (3)	C16—H16	0.9500
C4—H4	0.9500	C17—C18	1.494 (3)
C5—C6	1.395 (3)	C17—H17A	0.9900
C5—C7	1.506 (3)	C17—H17B	0.9900
C6—N1	1.332 (3)	C18—C19	1.528 (3)
C6—H6	0.9500	C18—H18A	0.9900
C7—C8	1.511 (3)	C18—H18B	0.9900
C7—H7A	0.9900	C19—C20	1.480 (4)
C7—H7B	0.9900	C19—H19A	0.9900
C8—C9	1.525 (3)	C19—H19B	0.9900
C8—H8A	0.9900	C20—H20A	0.9800
C8—H8B	0.9900	C20—H20B	0.9800
C9—C10	1.474 (4)	C20—H20C	0.9800
C9—H9A	0.9900	N1—Zn1	2.1007 (16)
C9—H9B	0.9900	N2—Zn1	2.0877 (17)
C10—H10A	0.9800	O1—Zn1	2.0416 (14)
C10—H10B	0.9800	O3—Zn1	2.0443 (14)
C10—H10C	0.9800	O1W—Zn1	1.9825 (14)
C11—O4	1.243 (2)	O1W—H1A	0.8498
C11—O3	1.264 (2)	O1W—H1B	0.8440
C11—C12	1.521 (3)	O2W—H2A	0.8514
C12—N2	1.341 (3)	O2W—H2B	0.8510
C12—C13	1.377 (3)		
			
O2—C1—O1	126.36 (19)	C15—C14—H14	120.0
O2—C1—C2	117.37 (18)	C16—C15—C14	117.25 (19)
O1—C1—C2	116.27 (17)	C16—C15—C17	120.7 (2)
N1—C2—C3	121.57 (19)	C14—C15—C17	122.0 (2)
N1—C2—C1	115.64 (17)	N2—C16—C15	122.97 (19)
C3—C2—C1	122.79 (18)	N2—C16—H16	118.5
C2—C3—C4	118.9 (2)	C15—C16—H16	118.5
C2—C3—H3	120.6	C18—C17—C15	114.50 (19)
C4—C3—H3	120.6	C18—C17—H17A	108.6
C5—C4—C3	120.3 (2)	C15—C17—H17A	108.6
C5—C4—H4	119.9	C18—C17—H17B	108.6
C3—C4—H4	119.9	C15—C17—H17B	108.6
C4—C5—C6	116.93 (19)	H17A—C17—H17B	107.6
C4—C5—C7	122.71 (19)	C17—C18—C19	113.5 (2)
C6—C5—C7	120.36 (19)	C17—C18—H18A	108.9
N1—C6—C5	122.96 (19)	C19—C18—H18A	108.9
N1—C6—H6	118.5	C17—C18—H18B	108.9
C5—C6—H6	118.5	C19—C18—H18B	108.9
C5—C7—C8	115.23 (19)	H18A—C18—H18B	107.7
C5—C7—H7A	108.5	C20—C19—C18	114.3 (2)
C8—C7—H7A	108.5	C20—C19—H19A	108.7
C5—C7—H7B	108.5	C18—C19—H19A	108.7
C8—C7—H7B	108.5	C20—C19—H19B	108.7
H7A—C7—H7B	107.5	C18—C19—H19B	108.7
C7—C8—C9	113.9 (2)	H19A—C19—H19B	107.6
C7—C8—H8A	108.8	C19—C20—H20A	109.5
C9—C8—H8A	108.8	C19—C20—H20B	109.5
C7—C8—H8B	108.8	H20A—C20—H20B	109.5
C9—C8—H8B	108.8	C19—C20—H20C	109.5
H8A—C8—H8B	107.7	H20A—C20—H20C	109.5
C10—C9—C8	114.8 (3)	H20B—C20—H20C	109.5
C10—C9—H9A	108.6	C6—N1—C2	119.38 (17)
C8—C9—H9A	108.6	C6—N1—Zn1	129.26 (14)
C10—C9—H9B	108.6	C2—N1—Zn1	111.10 (13)
C8—C9—H9B	108.6	C12—N2—C16	119.00 (17)
H9A—C9—H9B	107.6	C12—N2—Zn1	112.83 (13)
C9—C10—H10A	109.5	C16—N2—Zn1	128.17 (14)
C9—C10—H10B	109.5	C1—O1—Zn1	115.66 (13)
H10A—C10—H10B	109.5	C11—O3—Zn1	116.22 (12)
C9—C10—H10C	109.5	Zn1—O1W—H1A	111.6
H10A—C10—H10C	109.5	Zn1—O1W—H1B	110.8
H10B—C10—H10C	109.5	H1A—O1W—H1B	110.4
O4—C11—O3	125.56 (18)	H2A—O2W—H2B	108.2
O4—C11—C12	117.92 (17)	O1W—Zn1—O1	106.09 (6)
O3—C11—C12	116.52 (17)	O1W—Zn1—O3	102.80 (6)
N2—C12—C13	122.02 (18)	O1—Zn1—O3	151.11 (6)
N2—C12—C11	114.71 (17)	O1W—Zn1—N2	104.39 (6)
C13—C12—C11	123.26 (18)	O1—Zn1—N2	92.68 (6)
C12—C13—C14	118.69 (19)	O3—Zn1—N2	79.70 (6)
C12—C13—H13	120.7	O1W—Zn1—N1	103.25 (6)
C14—C13—H13	120.7	O1—Zn1—N1	79.91 (6)
C13—C14—C15	120.04 (19)	O3—Zn1—N1	93.96 (6)
C13—C14—H14	120.0	N2—Zn1—N1	152.36 (6)
			
O2—C1—C2—N1	178.32 (18)	C1—C2—N1—Zn1	−6.2 (2)
O1—C1—C2—N1	−2.8 (3)	C13—C12—N2—C16	−1.0 (3)
O2—C1—C2—C3	−2.0 (3)	C11—C12—N2—C16	177.81 (17)
O1—C1—C2—C3	176.9 (2)	C13—C12—N2—Zn1	179.31 (15)
N1—C2—C3—C4	−0.3 (3)	C11—C12—N2—Zn1	−1.9 (2)
C1—C2—C3—C4	−179.9 (2)	C15—C16—N2—C12	−0.2 (3)
C2—C3—C4—C5	0.8 (3)	C15—C16—N2—Zn1	179.46 (15)
C3—C4—C5—C6	−0.5 (3)	O2—C1—O1—Zn1	−170.40 (17)
C3—C4—C5—C7	179.7 (2)	C2—C1—O1—Zn1	10.8 (2)
C4—C5—C6—N1	−0.3 (3)	O4—C11—O3—Zn1	179.89 (16)
C7—C5—C6—N1	179.44 (19)	C12—C11—O3—Zn1	0.5 (2)
C4—C5—C7—C8	−45.1 (3)	C1—O1—Zn1—O1W	89.95 (15)
C6—C5—C7—C8	135.1 (2)	C1—O1—Zn1—O3	−90.86 (18)
C5—C7—C8—C9	−164.3 (2)	C1—O1—Zn1—N2	−164.24 (14)
C7—C8—C9—C10	−59.7 (4)	C1—O1—Zn1—N1	−11.06 (14)
O4—C11—C12—N2	−178.46 (18)	C11—O3—Zn1—O1W	101.39 (14)
O3—C11—C12—N2	0.9 (3)	C11—O3—Zn1—O1	−77.81 (18)
O4—C11—C12—C13	0.4 (3)	C11—O3—Zn1—N2	−1.18 (14)
O3—C11—C12—C13	179.76 (19)	C11—O3—Zn1—N1	−154.05 (14)
N2—C12—C13—C14	1.1 (3)	C12—N2—Zn1—O1W	−99.05 (13)
C11—C12—C13—C14	−177.59 (18)	C16—N2—Zn1—O1W	81.32 (17)
C12—C13—C14—C15	−0.1 (3)	C12—N2—Zn1—O1	153.59 (13)
C13—C14—C15—C16	−1.0 (3)	C16—N2—Zn1—O1	−26.04 (17)
C13—C14—C15—C17	177.75 (19)	C12—N2—Zn1—O3	1.66 (13)
C14—C15—C16—N2	1.1 (3)	C16—N2—Zn1—O3	−177.97 (17)
C17—C15—C16—N2	−177.61 (19)	C12—N2—Zn1—N1	80.37 (19)
C16—C15—C17—C18	−112.4 (3)	C16—N2—Zn1—N1	−99.3 (2)
C14—C15—C17—C18	68.9 (3)	C6—N1—Zn1—O1W	78.55 (18)
C15—C17—C18—C19	175.5 (2)	C2—N1—Zn1—O1W	−95.41 (14)
C17—C18—C19—C20	63.4 (4)	C6—N1—Zn1—O1	−177.13 (18)
C5—C6—N1—C2	0.9 (3)	C2—N1—Zn1—O1	8.90 (13)
C5—C6—N1—Zn1	−172.66 (15)	C6—N1—Zn1—O3	−25.60 (18)
C3—C2—N1—C6	−0.6 (3)	C2—N1—Zn1—O3	160.44 (13)
C1—C2—N1—C6	179.13 (17)	C6—N1—Zn1—N2	−100.9 (2)
C3—C2—N1—Zn1	174.08 (16)	C2—N1—Zn1—N2	85.17 (19)

### Hydrogen-bond geometry (Å, °)

**Table:**

*D*—H···*A*	*D*—H	H···*A*	*D*···*A*	*D*—H···*A*
O2W—H2B···O4	0.85	1.94	2.780 (2)	172
O2W—H2A···O4^i^	0.85	2.02	2.849 (2)	165
O1W—H1B···O2W^ii^	0.84	1.80	2.638 (2)	175
O1W—H1A···O2^iii^	0.85	1.79	2.636 (2)	174

Symmetry codes: (i) −*x*+1, −*y*+1, −*z*; (ii) −*x*+2, −*y*+1, −*z*; (iii) −*x*+2, −*y*, −*z*.
